# Up-Front Immune Checkpoint Inhibitors in Patients with Multiple Synchronous Primary Tumors, Including dMMR/MSI-H Gastrointestinal Malignancies: A Case Series and Review of the Literature

**DOI:** 10.3390/curroncol33100594

**Published:** 2026-10-01

**Authors:** Angela Damato, Martina Rotolo, Nicoletta Sparacino, Eleonora Borghi, Giacomo Santandrea, Angelina Filice, Mario Larocca, Carmine Pinto

**Affiliations:** 1 Medical Oncology, Fondazione IRCCS Ca’ Granda, Ospedale Maggiore Policlinico, 20122 Milan, Italy; 2 Medical Oncology Unit, Azienda USL-IRCCS of Reggio Emilia, 42123 Reggio Emilia, Italyeleonora.borghi@ausl.re.it (E.B.); carmine.pinto@ausl.re.it (C.P.); 3 Department of Oncology and Hematology, Modena University Hospital, University of Modena and Reggio Emilia, 41124 Modena, Italy; 4 Pathology Unit, Azienda USL-IRCCS of Reggio Emilia, 42123 Reggio Emilia, Italy; 5 Nuclear Medicine Unit, Azienda USL-IRCCS of Reggio Emilia, 42123 Reggio Emilia, Italy

**Keywords:** synchronous primary tumors, mismatch repair deficiency, immunotherapy, frail patients, non-operative management

## Abstract

The management of patients with multiple synchronous primary tumors remains challenging, particularly in individuals who are not candidates for surgery. While immune checkpoint inhibitors (ICIs) have demonstrated remarkable efficacy in deficient mismatch repair (dMMR)/microsatellite instability-high (MSI-H) tumors, evidence supporting their use as a non-operative strategy in patients with multiple malignancies is limited, as this population is excluded from clinical trials. We report three cases of patients with synchronous primary tumors, including gastrointestinal malignancies with dMMR status, treated with up-front ICIs. Treatment strategies were individualized based on the patient’s clinical condition, tumor burden, and molecular profile. All patients achieved meaningful and durable responses with long-term survival exceeding two to four years, supporting the feasibility of a non-operative strategy and organ preservation in carefully selected patients, including the frail subgroup population. Immunotherapy-based approaches were well tolerated, with a manageable safety profile. Notably, none of our patients underwent surgical resection. However, these observations are hypothesis-generating and cannot establish the efficacy or equivalence of immunotherapy to standard surgical management. Our findings extend current evidence to a largely underrepresented population and support a personalized, multidisciplinary, response-adapted approach. Prospective studies are warranted to better define patient selection, optimal treatment duration, and long-term outcomes of non-operative strategies in this setting.

## Simple Summary

Patients with a concurrent diagnosis of multiple primary tumors present a particularly complex challenge in terms of determining the appropriate treatment, as these patients are generally excluded from clinical trials—especially if they have other comorbidities—due in part to the risks associated with surgery. Based on the histological and molecular characteristics of the tumor, immunotherapy may be a viable non-surgical option for these patients. We describe three patients with synchronous primary tumors, including gastrointestinal tumors in which the mismatch repair status is a therapeutic target, who received immunotherapy as an alternative to other up-front therapeutic approaches. All patients achieved significant and durable disease control, with complete responses at some tumor sites and long-term survival, suggesting that in carefully selected patients, immunotherapy may represent a valid option. Further prospective studies are needed to identify patients who might benefit most and to define the optimal treatment strategy.

## 1. Introduction

In locally advanced gastric/gastroesophageal junction adenocarcinoma (GEJ) and colon cancer settings, new biomarkers could guide treatment decisions. Several studies are underway to demonstrate the efficacy of immunotherapy as part of a non-surgical management strategy for these tumors, which currently involve perioperative chemotherapy with surgical resection [1] and surgery followed by adjuvant chemotherapy, respectively [2].

A deficiency in the mismatch repair system is detected in approximately 10% and 10–15% of non-metastatic gastric/GEJ adenocarcinomas and colon tumors, respectively [3,4]. In this context, many tumor neoantigens increase the activity of antitumor immune T cells, suggesting a tumor immune microenvironment that could favor anti-PD-1/PD-L1 antibody blockade [5]. Therefore, a better response could be achieved through early application of ICIs, particularly in dMMR/MSI-H tumors, across different tumor types [5,6].

The INFINITY phase II study [7] evaluated the efficacy and safety of tremelimumab plus durvalumab for three cycles as neoadjuvant (cohort 1) or definitive (cohort 2) treatment of resectable dMMR gastric/GEJ tumors. The primary endpoint was the pathological complete response (pCR) rate in cohort 1 and the clinical complete response (cCR) rate at 2 years in cohort 2, reaching 60% and 76%, respectively. Only 17% of patients experienced Grade 3 immune-related adverse events (irAEs), including colitis, pneumonitis, and liver toxicity.

As revealed by the phase II NEONIPIGA study [8], neoadjuvant nivolumab plus ipilimumab was associated with high pCR rates in resectable dMMR gastric/GEJ adenocarcinoma. In this study, 68% of patients were stage cT2-3N1, 28% were stage cT2-3N0, and one patient was mistakenly included at baseline due to metastasis. At a median follow-up of 14.9 months, 84% of patients completed the six planned treatment cycles, and 91% underwent surgery, achieving R0 surgical resection. Among all patients, 19% experienced Grade 3–4 AEs, making this combination overall safe and manageable.

In the phase II NICHE-2 study [9], stage II and III dMMR/MSI-H colon cancer patients were treated with two cycles of nivolumab plus ipilimumab as neoadjuvant therapy. Overall, 115 enrolled patients underwent surgery with R0 resection. Ninety-eight percent underwent timely surgery, while only 2% experienced a delay due to AEs. A pathological response rate of 99% and a pCR of 68% were reported. At a median follow-up of 36.5 months, no disease recurrence was reported, and a 3-year disease-free survival rate was 100%. Grade > 3 AEs occurred in only 4% of patients.

Furthermore, the results of the phase II NICHE-3 study [10] showed that in 59 patients with locally advanced dMMR colon cancer (63% in stage III), two doses of neoadjuvant nivolumab and relatlimab achieved tumor-free surgical margins and pCR in 100% and 68% of cases, respectively. Grade 3–4 AEs occurred in 10% of cases, causing delays in surgery. In this context, liquid biopsy—particularly the analysis of circulating tumor DNA (ctDNA)—is emerging as a promising tool for the early monitoring of treatment response, the assessment of residual disease, and its molecular recharacterization. Its integration into response-adaptive strategies could therefore facilitate more personalized treatment decisions, including the identification of who might benefit from continuing or discontinuing treatment [11].

However, patients with synchronous primary tumors and frailty are systematically excluded from clinical trials, leaving a major evidence gap in real-world decision-making. Accordingly, real-world evidence is particularly relevant for defining treatment strategies in patients whose clinical complexity prevents direct extrapolation from prospective trials. In this context, we describe three patients with synchronous primary malignancies of which some with dMMR/MSI-H status, who received an up-front immunotherapy-based approach, with particular attention to treatment feasibility, disease control, and the role of a multidisciplinary, response-adapted approach.

## 2. Case Presentation

### 2.1. Patient 1

**Figure 1:**
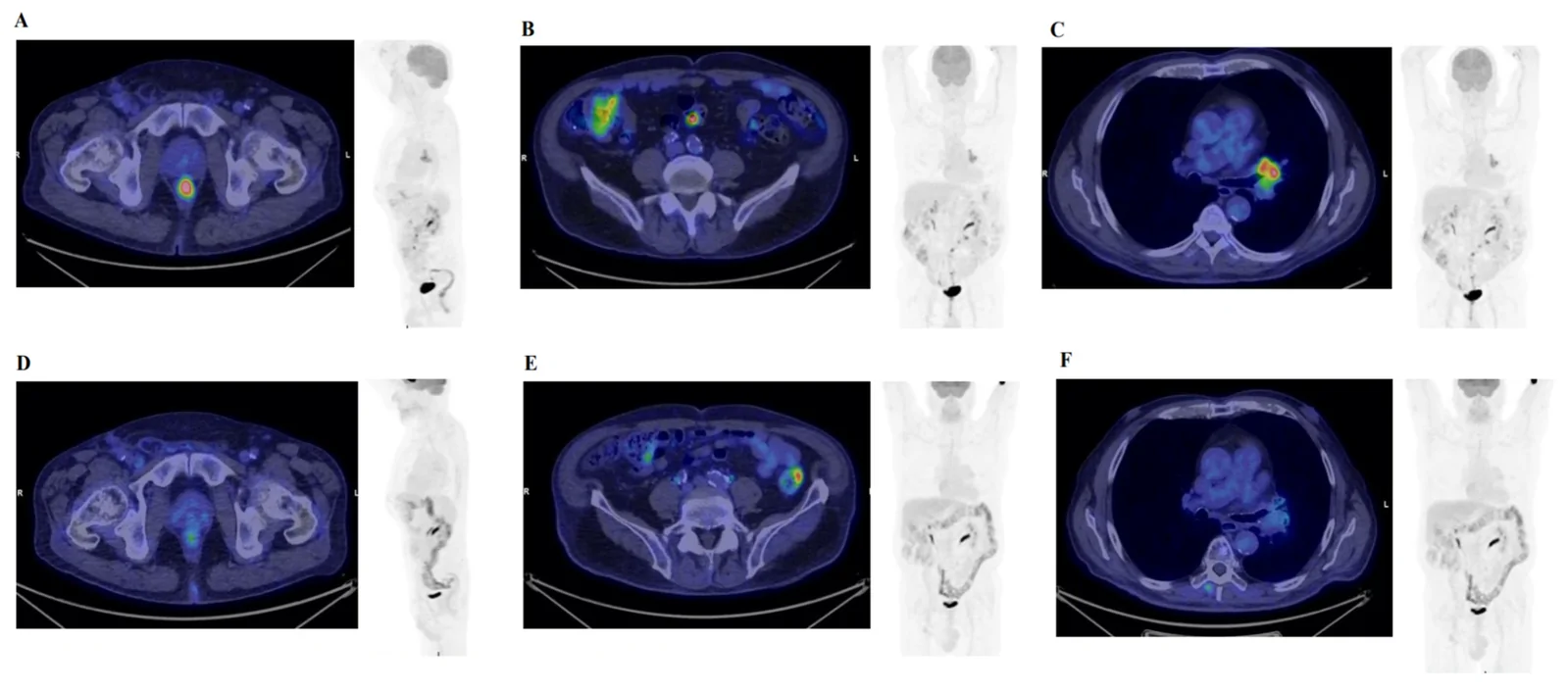
18F-FDG PET scan depiction of Patient 1 with three synchronous tumors at diagnosis and after combined treatment strategies. Panels (**A**–**C**) show the baseline 18F-FDG PET uptake of low-differentiated squamous cell carcinoma of the anorectal mucosa (cT3cN2cM0), an infiltrating adenocarcinoma of the hepatic colon flexure (cT3cN0cM0, dMMR IHC), and a left lung squamous cell carcinoma (cT2cN2cM0), respectively. Panels (**D**–**F**) depict the follow-up 18F-FDG PET scan after four cycles of chemotherapy (carboplatin and paclitaxel) combined with pembrolizumab, followed by pembrolizumab maintenance and SBRT to the residual pulmonary lesion. No residual metabolic disease was observed at the three primary tumor sites.

Patient 1, in their 70s, is a heavy smoker with several comorbidities (type II diabetes mellitus, peripheral vascular disease, ischemic heart disease, atrial fibrillation, and chronic obstructive pulmonary disease) and Eastern Cooperative Oncology Group (ECOG) Performance Status (PS) of 2. Due to the appearance of a perianal nodular lesion and rectal bleeding, the patient underwent a surgical examination and colonoscopy, which revealed: (i) anorectal low-differentiated squamous cell carcinoma, stage III (cT3N2M0); (ii) colon hepatic flexure infiltrating adenocarcinoma, stage II-cT3N0M0 (dMMR [MLH1 and PMS2 loss], BRAF V600E, PD-L1 CPS 20). Additionally, the radiological chest CT scan displayed a synchronous left lung nodule and mediastinal lymph nodes; a bronchoscopic biopsy confirmed squamous cell carcinoma, stage III-cT2N2M0 (PD-L1 TPS 3%) (Figure 1A–C).

**Figure 2:**
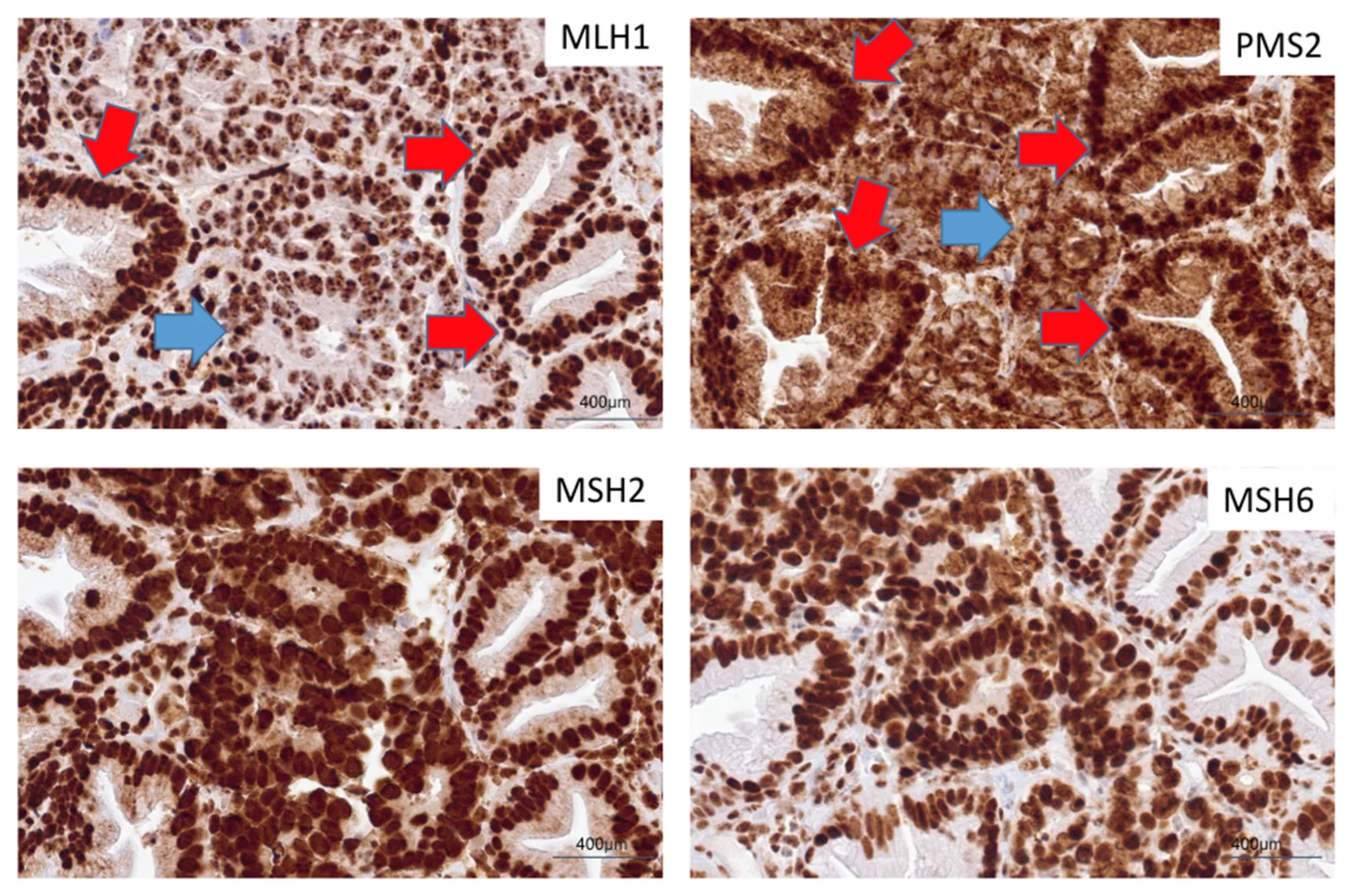
Immunohistochemistry of mismatch repair proteins: poorly differentiated adenocarcinoma (blue arrow) showing loss of MLH1 and PMS2 expression and intact MSH2 and MSH6 expression. The red arrows indicate non-neoplastic intestinal glands (for comparison).

Given the presence of three synchronous malignancies and severe cardiovascular comorbidities, the patient was deemed unfit for surgery by the multidisciplinary tumor board, and according to dMMR status (Figure 2), the patient was a candidate for an up-front systemic treatment based on platinum derivatives (carboplatin) and paclitaxel in combination with an anti-PD-1 antibody, pembrolizumab. Four cycles of chemotherapy and immunotherapy were administered, resulting in a partial response of the disease in both the lungs and the intestines with normalization of metabolic uptake. Therefore, treatment with pembrolizumab was continued, resulting in a clinical complete response (cCR) in the anal canal and a partial response in the lungs and colon, always according to these evaluation criteria.

Given the persistence of residual pulmonary disease, stereotactic body radiotherapy (SBRT) was subsequently administered to the lung lesion as a local consolidative treatment. After 11 cycles of pembrolizumab, approximately one year after treatment initiation, immunotherapy was discontinued because of Grade 3 interstitial pneumonia and Grade 2 diarrhea, requiring hospitalization, oxygen supplementation, and high-dose corticosteroid therapy, with subsequent resolution of the immune-related adverse events.

Approximately five months after treatment discontinuation, colonoscopy and 18F-FDG PET scan showed no evidence of residual disease at any of the three tumor sites. At three and a half years from diagnosis, the patient remains alive without evidence of disease recurrence (Figure 1D–F). The tumor diagnosis, instrumental evaluation, treatments, and disease response are depicted in Table 1 and Figure 7A.

### 2.2. Patient 2

**Figure 3:**
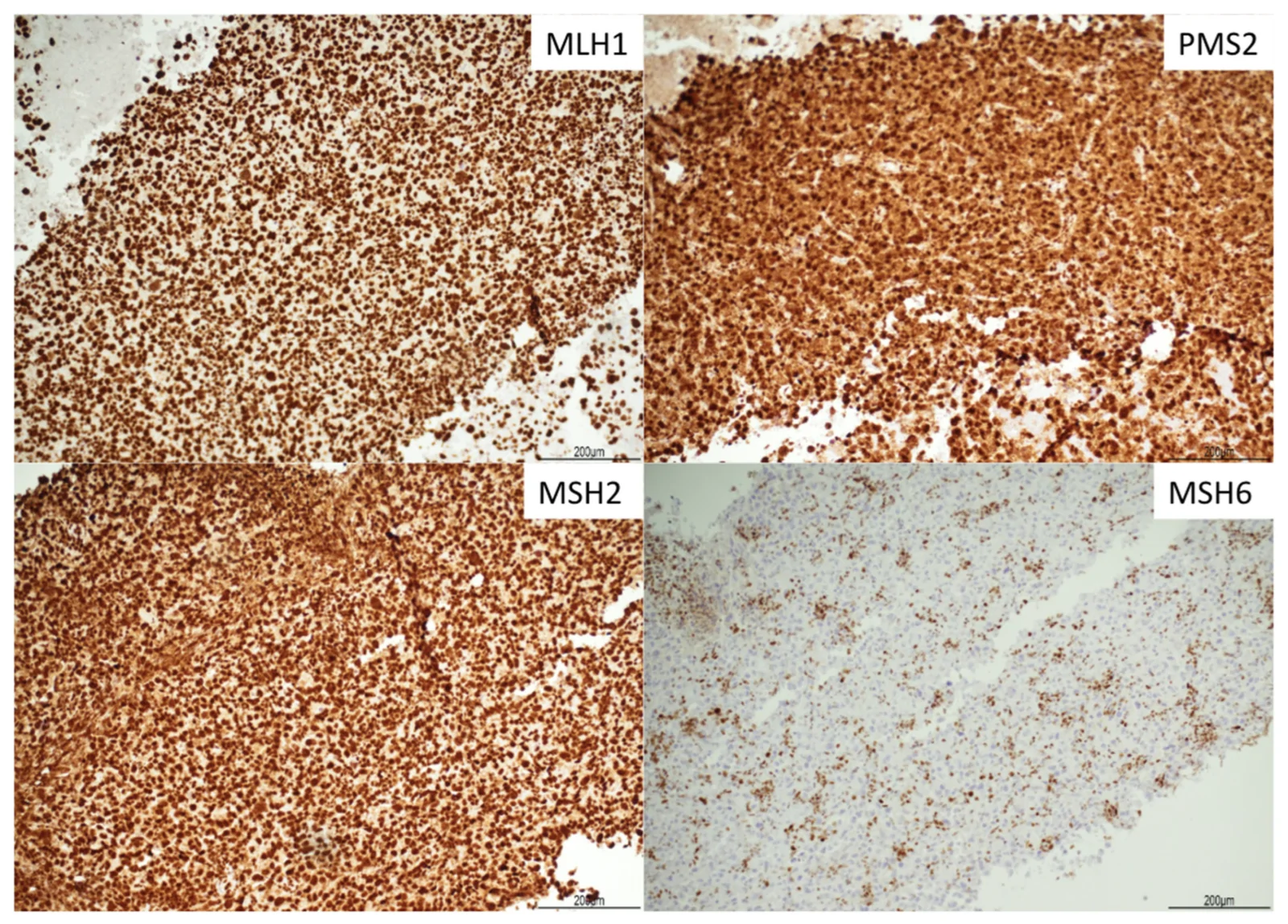
Immunohistochemistry of mismatch repair proteins: preserved expression of MLH1, PMS2, and MSH2 with loss of MSH6 expression, consistent with a dMMR phenotype.

Patient 2, in his 70s, is a former smoker with emphysematous changes and chronic obstructive pulmonary disease with colon diverticulosis and ECOG PS 1. Due to the onset of worsening epigastric pain, the patient underwent a CT scan, which revealed liver metastases and lateral cervical and abdominal lymph nodes, as well as a single 4-cm nodular lesion in the right upper lobe of the lung. A subsequent colonoscopy confirmed the presence of a villous lesion in the distal ascending colon, histologically consistent with adenocarcinoma (cT3,cN2,cM1, dMMR [MSH6 loss]) (Figure 3).

An Endobronchial Ultrasound (EBUS) with biopsy was performed, revealing stage IB primary pulmonary adenocarcinoma (cT2a, cN0, *KRAS G12C*, PD-L1 TPS 50%).

**Figure 4:**
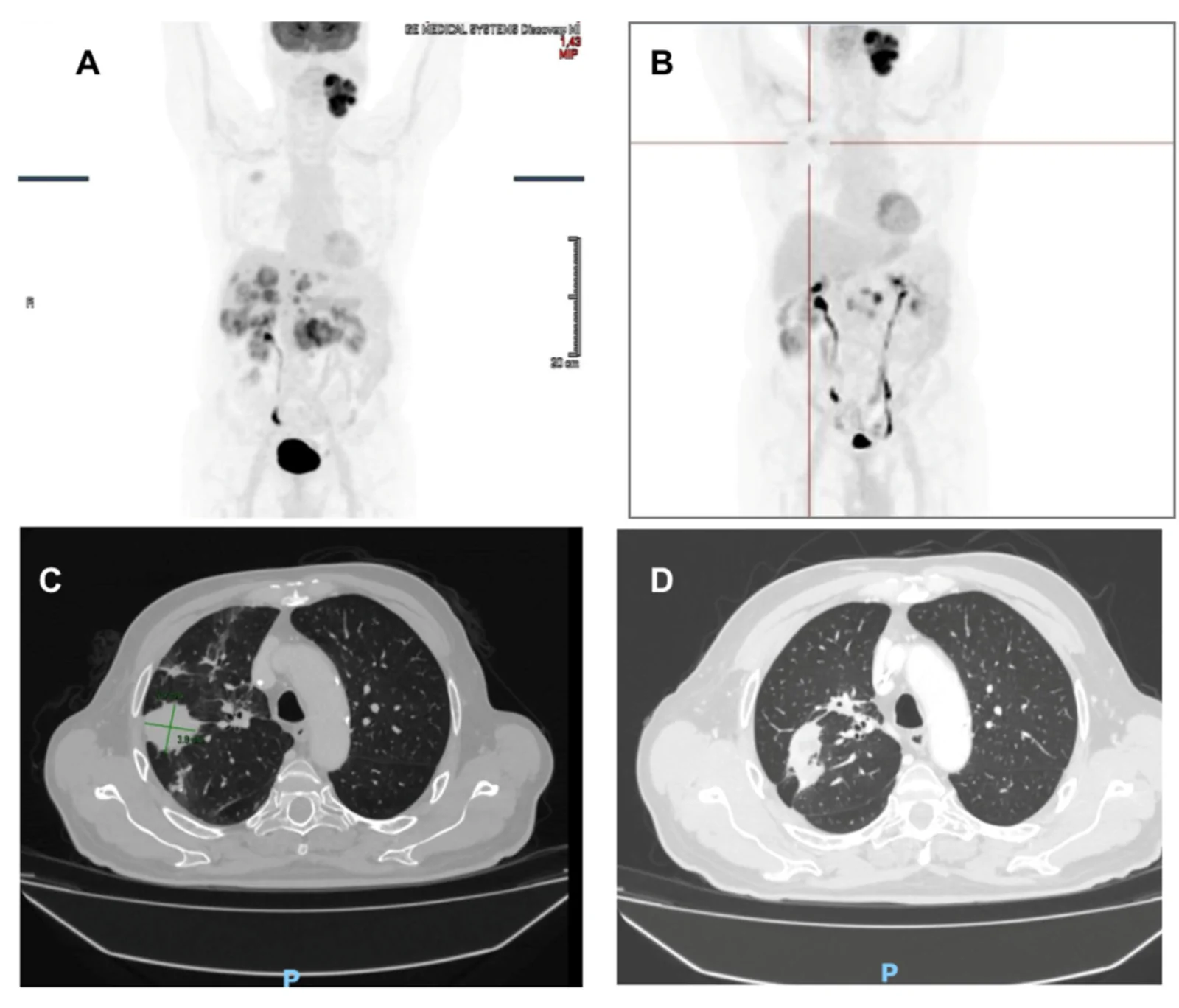
18F-FDG PET and CT scan of Patient 2 at diagnosis and at the 3-month follow-up: Panel (**A**) shows the baseline 18F-FDG PET uptake of adenocarcinoma of the right colon with multiple liver metastases and lateral cervical and abdominal lymph nodes (cT3cN2cM1, dMMR (IHC)), and a right lung adenocarcinoma (cT2cN0cM0), respectively. Panel (**B**) depicts the PET scan after 6 cycles of anti-PD-1 antibody, pembrolizumab, and SBRT of the primary lung cancer, in which there is a complete response in the liver and partial response in the lymph node metastases. Panel (**C**) shows the baseline CT scan with a right lung primary tumor, while Panel (**D**) shows it 3 months after radiation therapy.

After multidisciplinary discussion, given the presence of two synchronous tumors, one of which was stage IV due to the presence of liver metastases, PD-L1 positivity, and dMMR status, the patient was a candidate for an up-front systemic treatment with pembrolizumab and stereotactic radiotherapy to the pulmonary nodule (50 Gy dose in 5 fractions). Six cycles of immunotherapy were administered with an excellent response in the liver on CT scan, followed by normalization of PET uptake, stable disease of the lateral cervical lymph nodes, and a partial response in the primary colon cancer (Figure 4A,B). Three months after the end of radiotherapy, the primary lung tumor had regressed (Figure 4C,D), according to the RECIST criteria v1.1 [12].

Treatment with pembrolizumab is still ongoing, with a sustained response in each site of colon cancer and stable disease of the lung tumor, and no immune-related AEs were observed. To date, nearly two years after diagnosis, the patient is still alive, with no progression of the disease. The tumor diagnosis, instrumental evaluation, treatments, and disease response are depicted in Table 1 and Figure 7B.

### 2.3. Patient 3

**Figure 5:**
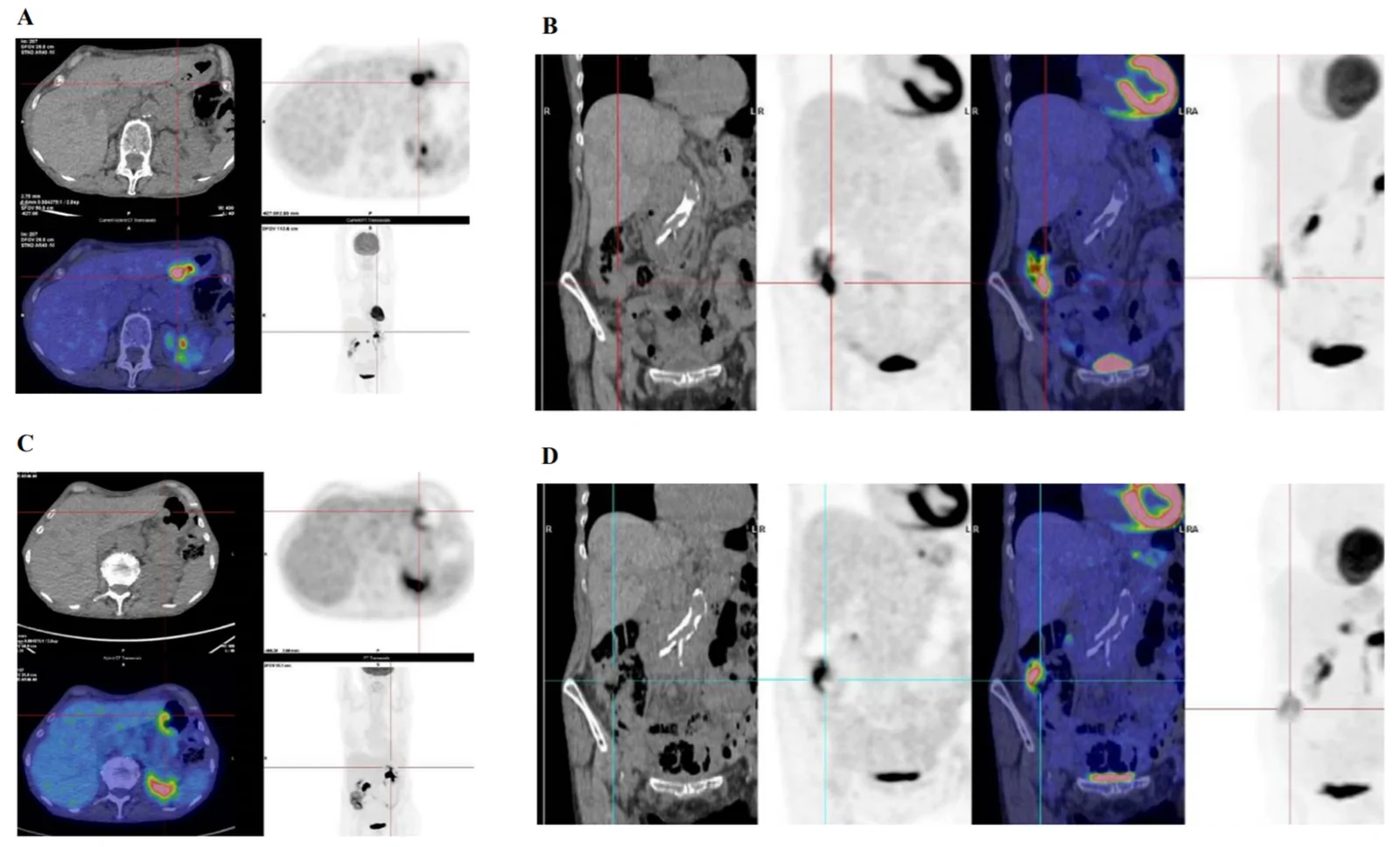
18F-FDG PET scan depiction of Patient 3 with two synchronous tumors at diagnosis and during immunotherapy treatment. Panels (**A**,**B**) show the baseline 18F-FDG PET uptake: (**A**) a low differentiated infiltrating adenocarcinoma of the gastric angulus (uT4uN2M0, dMMR IHC), (**B**) and stenotic right colon mucinous adenocarcinoma (cT3cN0cM0, dMMR IHC). Panels (**C**,**D**) depict the PET scan response after 3 years of anti-PD-1 antibody, pembrolizumab, with a slight gastric uptake in the antrum and pyloric sites compatible at endoscopy with a well-differentiated neuroendocrine tumor (**C**), and a tight residual ulcerated adenoma with diffuse high-grade dysplasia in the ascending colon (**D**). Pembrolizumab treatment is still ongoing, and the patient continues to refuse surgical approaches.

Patient 3, in their 70s, is in good clinical condition (ECOG PS 0) with a history of thyroidectomy for stage III papillary carcinoma on hormone replacement therapy, and hysterectomy with bilateral adnexectomy for uterine prolapse. For epigastric pain, weight loss, and sideropenic anemia, a gastroscopy and colonoscopy with biopsies were performed. The diagnosis was low-differentiated infiltrating adenocarcinoma of the gastric angulus (uT4N2M0, stage III, dMMR with isolated PMS2 loss, HER2 unamplified, PD-L1 CPS 1, Figure 5A) and a synchronous right colon mucinous adenocarcinoma (cT3cN0cM0, dMMR [MLH1 and PMS2 loss], *BRAF V600E*) (Figure 5B).

**Figure 6:**
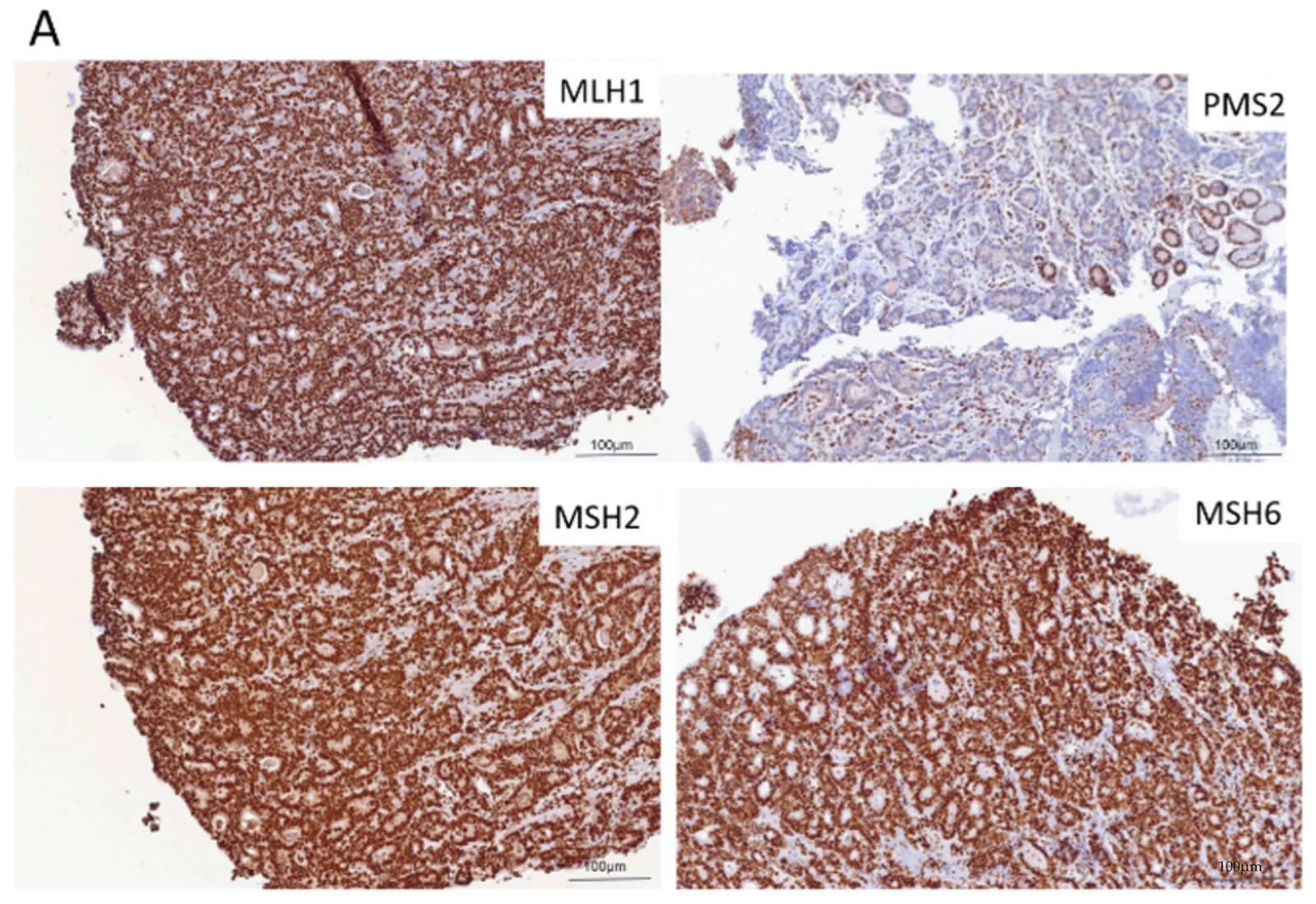

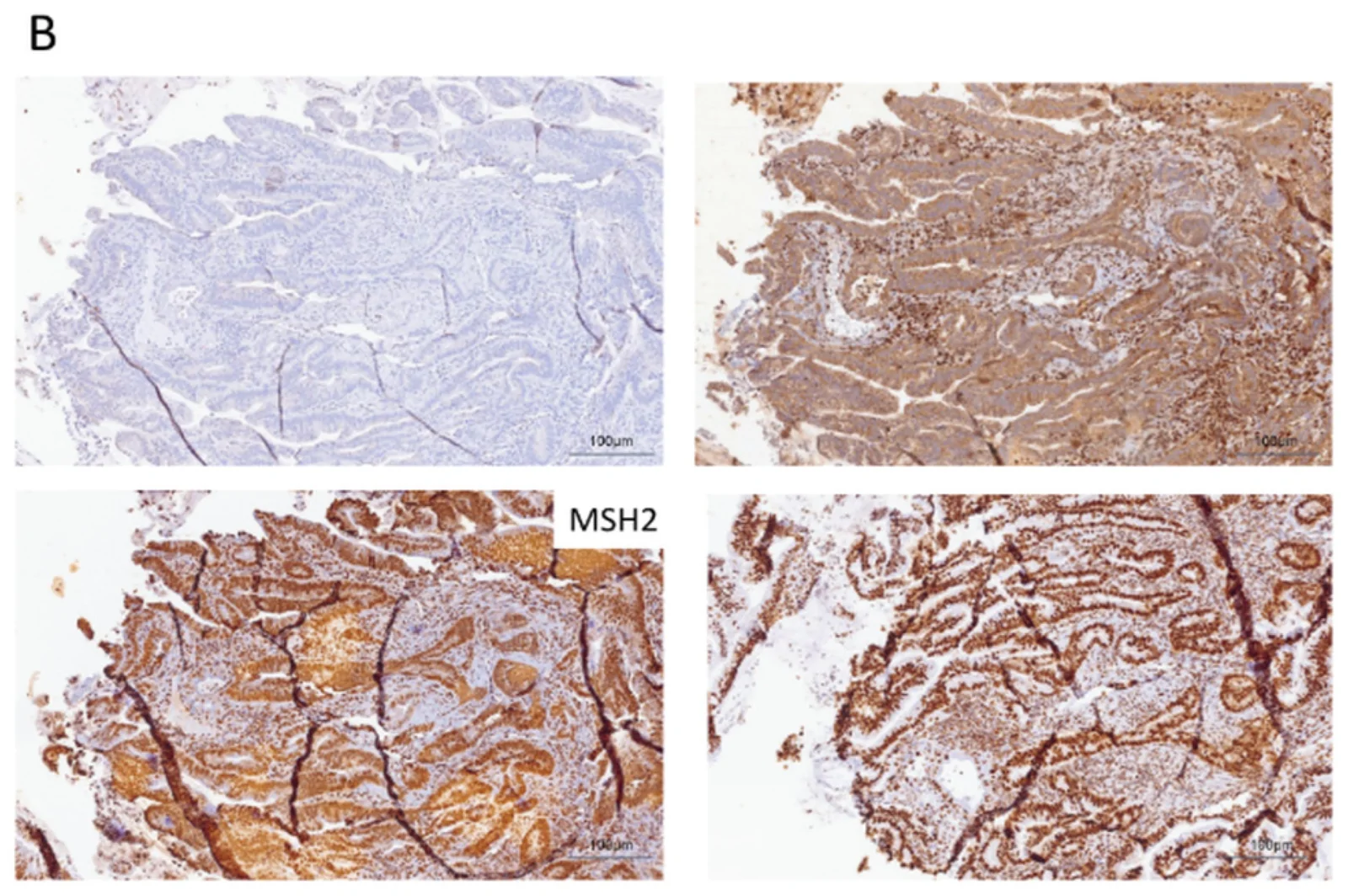
Immunohistochemistry of mismatch repair proteins: (**A**) Poorly differentiated gastric adenocarcinoma with intact expression of MLH1, isolated PMS2 loss, MSH2 and MSH6 expression. (**B**) Moderately differentiated adenocarcinoma with loss of MLH1 and PMS2 expression and intact MSH2 and MSH6 expression.

**Figure 7:**
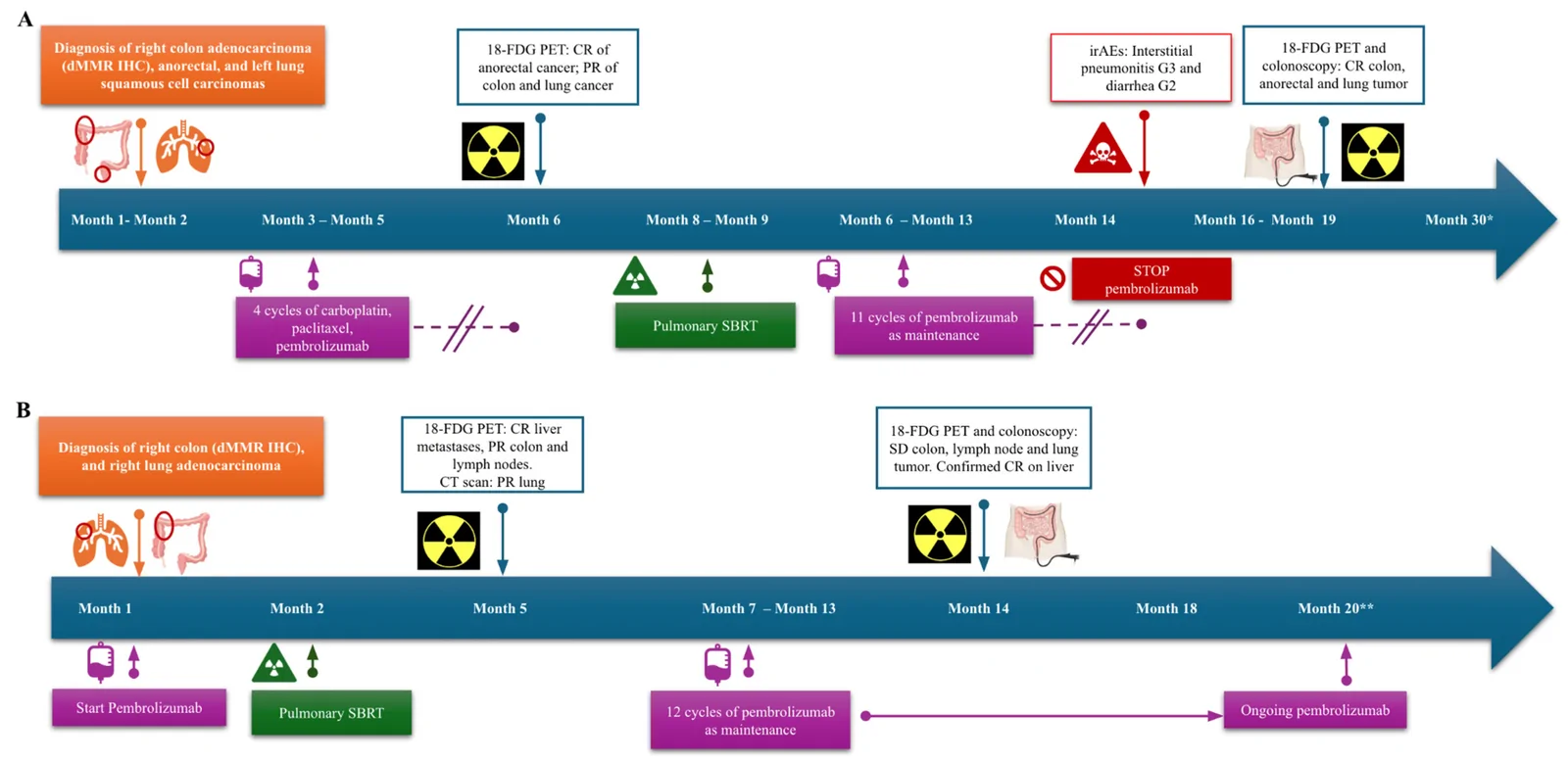

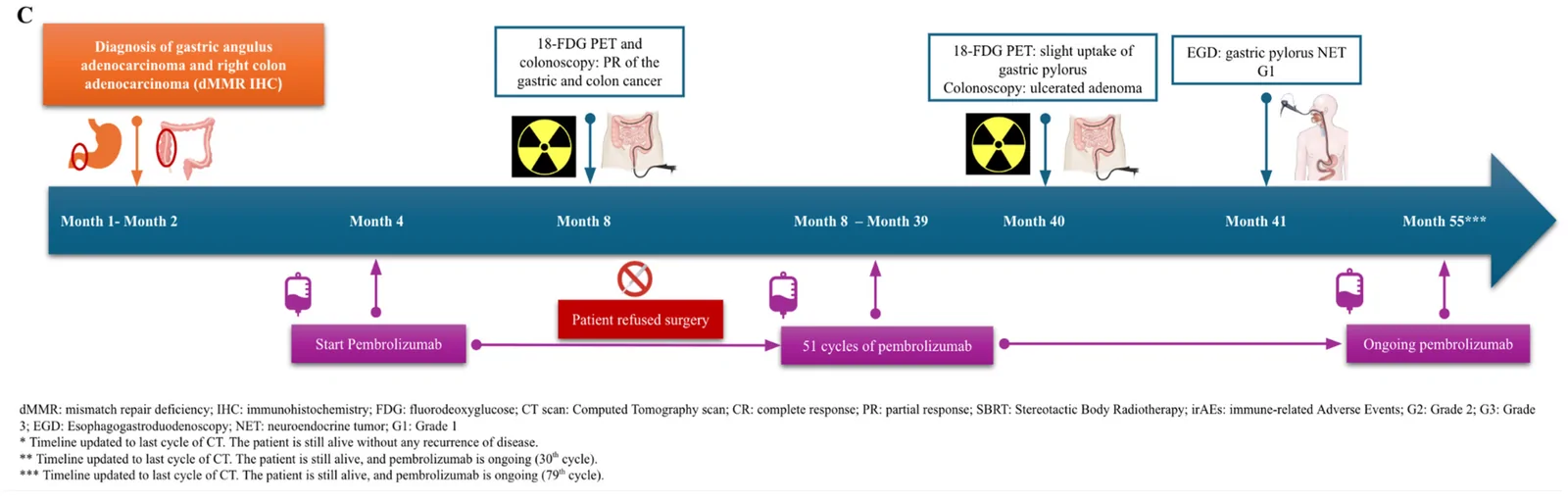
Timeline of tumor diagnosis, instrumental evaluation, treatments, and response of Patients 1, 2, and 3. In Panel (**A**), Patient 1 was diagnosed with 3 synchronous locally advanced tumors (right colon, anal canal, and left lung) and was treated with chemoimmunotherapy, maintenance immunotherapy, and stereotactic radiotherapy, with a subsequent complete response in each tumor site. No surgery was performed due ECOG PS and comorbidities. In Panel (**B**), Patient 2 was diagnosed with 2 synchronous tumors (advanced right colon and right lung) and was treated with immunotherapy and stereotactic radiotherapy, with a good response in each tumor site. No surgery was performed due to the stage of the disease. In Panel (**C**), Patient 3 was diagnosed with 2 synchronous tumors (gastric angulus and right colon) and started immunotherapy, which continued over 3 years and is still ongoing, reaching a partial response in the right colon and a complete response in gastric cancer. At any time, Patient 3 refused surgery.

Since the dMMR for both neoplasms (Figure 6), systemic treatment with pembrolizumab was started, resulting in sustained disease control for more than 3 years. (Figure 5C,D). After a multidisciplinary discussion, despite the patient’s age, surgery was repeatedly proposed, but was declined. Nearly 3 years after the start of well-tolerated immunotherapy, the colonoscopy showed a tight ulcerated adenoma with diffuse high-grade dysplasia in the ascending colon, a possible persistence of the disease. The 18F-FDG PET revealed slight gastric uptake in the antrum-pyloric site, suspected of local recurrence (Figure 5C); the gastroscopy, however, documented a complete endoscopic response and displayed a well-differentiated neuroendocrine tumor G1 in the gastric antrum. To date, after 4.5 years, the patient is still alive, and the immunotherapy is ongoing and well-tolerated. The tumor diagnosis, instrumental evaluation, treatments, and disease response are depicted in Table 1 and Figure 7C. The subsequent gastric neuroendocrine tumor was considered a distinct neoplastic lesion, and its response to pembrolizumab could not be assessed because MMR status was not available.

## 3. Discussion

These three case reports demonstrate that in patients with multiple synchronous primary tumors, some with dMMR status, the up-front systemic ICIs could lead to a favorable outcome and avoid potential risks associated with surgery. Patient 1 was considered unsuitable for surgery due to clinical condition and comorbidities; Patient 2 had metastatic disease at baseline and was consequently not a candidate for surgery; nevertheless, immunotherapy produced a meaningful and durable response. Patient 3, although in good clinical condition, refused surgery. Therefore, a non-surgical strategy was adopted in all three clinical cases. The clinical scenarios were heterogeneous, reflecting the complexity of synchronous primary malignancies in real-world practice. Therefore, our observations should be interpreted as hypothesis-generating rather than as evidence supporting a uniform treatment strategy. Deficiency in the MMR system leads to the accumulation of insertion–deletion mutations with a markedly elevated tumor mutational burden (TMB) and the generation of numerous frameshift-derived neoantigens. These molecular features promote a highly inflamed tumor microenvironment characterized by dense CD8+ T-cell infiltration, interferon-γ-driven signaling, and compensatory upregulation of immune checkpoints such as PD-1 and PD-L1, providing the mechanistic basis for the responsiveness of dMMR/MSI-H tumors to ICIs [13].

Therefore, during the diagnostic phase, it is mandatory to assess the patient’s general condition to decide the optimal therapeutic strategy, limiting risks, even if opting for systemic treatment, such as ICI monotherapy or in combination with chemotherapy.

In Patient 1, carboplatin plus paclitaxel was selected based on the evidence from the InterAAct trial supporting this regimen in advanced anal squamous cell carcinoma [14]. The concomitant use of pembrolizumab is also supported by the antitumor activity of immune checkpoint inhibitors reported in advanced anal SCC, which may occur irrespective of dMMR/MSI-H status [15].

Similarly, the response of the non-dMMR lung tumors in Patients 1 and 2 may be explained by the established activity of pembrolizumab in NSCLC, which is primarily related to PD-L1 expression and tumor immune biology rather than MMR status [16,17,18].

Our cases extend current evidence to a largely unrepresented population in the clinical trials. The NEONIPIGA [8] and NICHE-2 [9] studies enrolled patients with good ECOG Performance Status and younger age (median age 60 years) with stage II/III dMMR gastric or colon cancer, excluding frail patients who generally have high surgery-related mortality. For this subcategory of patients, an individualized conservative approach may be considered when standard surgical management is not feasible or is declined.

As is known, immunotherapy in dMMR tumors has proven to be extremely effective in rectal cancer as a non-surgical management strategy. In the initial report by Cercek et al. [19], 12 patients with locally advanced dMMR rectal cancer who had completed 6 months of dostarlimab achieved a clinical complete response (cCR), with no patients requiring chemoradiotherapy or surgery at the time of the report. In the subsequent 2024 update of the study [20], 41 of the 47 enrolled patients had completed the planned 6-month treatment, and all 41 achieved a clinical complete response (cCR). Among these patients, 20 achieved a sustained cCR, with a median follow-up of 28.9 months from treatment initiation (95% CI, 22.9–37.1). No patients required additional therapy, and no local or distant disease recurrence was observed.

Although the literature data derive from small-scale, non-randomized, single-arm phase II studies, the neoadjuvant strategy with ICIs in dMMR tumors appears to be effective and manageable, suggesting that this approach may allow frail patients to avoid further treatments, especially invasive surgical procedures. A non-surgical management in a specific subgroup of patients can be envisaged, despite the presence of synchronous primary tumors, as in our three clinical cases.

The decision-making phase is crucial for discussing with all professionals involved in the multidisciplinary setting and assessing the potential short- and long-term adverse events that both surgery and immunotherapy may entail, thus choosing the best option for the patient.

In the metastatic setting, the KEYNOTE-177 trial established pembrolizumab as a first-line treatment option for patients with MSI-H/dMMR metastatic colorectal cancer, demonstrating improved progression-free survival compared with chemotherapy [21]. These findings provide an important clinical context for the management of Patient 2, who presented with metastatic dMMR disease and received immune checkpoint inhibition as systemic treatment. Long-term follow-up data have further supported the durability of responses observed with first-line pembrolizumab in this molecularly defined population [22].

Another critical issue concerns assessing the dMMR status, its frequency in relation to the risk of developing synchronous tumors, and therefore the response to immunotherapy [23,24].

MSI is observed in approximately 10–15% of colorectal cancers and in a subset of gastric cancers [25], with sporadic MSI-H tumors frequently associated with MLH1 promoter hypermethylation. Some data from cohort studies suggest that the incidence of dMMR/MSI-H is significantly higher in patients with multiple tumors than in patients with single tumors [26]. Specifically, high-frequency MSI-H was found in patients with multiple tumors in the same organ, compared to low-frequency MSI more represented in multiple tumors of different districts [27,28].

This variability underlines the importance of site-specific molecular assessment, as therapeutic response depends on the precise immunogenomic profile of each tumor.

The presence of multiple synchronous dMMR tumors also suggests the existence of an underlying hereditary mismatch repair syndrome. Although most cases of dMMR/MSI-H tumors are sporadic, tumor-based MMR/MSI assessment represents an important strategy for identifying patients who may benefit from genetic counseling and germline testing, including those with tumor types not classically associated with Lynch syndrome [29]. In Patients 1 and 3, the *BRAF V600E* mutation in MLH1-deficient colorectal tumors supports the hypothesis of a sporadic pathway of MLH1 hypermethylation rather than Lynch syndrome. In Patient 3, the isolated loss of PMS2 in the gastric tumor and the isolated loss of MSH6 in Patient 2 led to germline testing, which did not identify any pathogenic variants.

Another point that remains unclear concerns the duration of immunotherapy. For both early- and advanced-stage colorectal cancer, this remains to be determined. However, in the KEYNOTE-177 and CheckMate 8HW studies, conducted in a metastatic setting, the duration of treatment was two years. Recent data from clinical practice suggest that, once a response is achieved, early discontinuation of treatment does not adversely affect progression-free survival or overall survival, regardless of whether the discontinuation is due to toxicity, surgery, or patient preference [30,31]. Liquid biopsy, particularly circulating tumor DNA, may represent a promising tool for identifying patients with sustained molecular response and guiding personalized treatment discontinuation [32].

Patients with multiple primary tumors are commonly excluded from clinical trials, limiting the evidence base for this subgroup. Early data from NICHE-3 [10], in which, among the 59 patients enrolled, two patients had synchronous colonic tumors and one of them had dMMR colon cancer and showed pCR and significant pathological response with ICIs, respectively, without unexpected toxicity, support the applicability of immunotherapy even in more complex settings. Our cases align with these findings, demonstrating that molecular features—particularly dMMR/MSI-H—remain strong predictors of benefit despite clinical complexity.

This case series has several limitations. First, the small number of patients and the retrospective descriptive nature of the analysis preclude any assessment of treatment efficacy or comparison with standard surgical management. Second, the clinical and oncological characteristics of the three patients were heterogeneous, including different tumor sites, disease stages, comorbidities, and treatment regimens. Third, in some cases, immunotherapy was administered in combination with chemotherapy or locoregional treatments, making it difficult to attribute disease control exclusively to immune checkpoint inhibition. Finally, the absence of systematic molecular characterization across all synchronous tumors limits the possibility of defining the contribution of tumor-specific biomarkers to treatment response. Therefore, these observations should be considered hypothesis-generating and primarily supportive of individualized multidisciplinary decision-making rather than as evidence for replacing standard surgical approaches.

In conclusion, our experiences highlight how a precision oncology approach focused on MMR/MSI testing can influence treatment decisions in patients with synchronous primary malignancies. By exploiting the high TMB, high neoantigen burden, and immune-inflammatory microenvironment characteristic of dMMR tumors, early administration of ICIs was associated with durable disease control and was integrated with personalized locoregional interventions. This strategy not only mitigates the risks associated with surgery but also offers the possibility of durable disease control.

Further prospective studies are needed to better define conservative immunotherapy-based treatment strategies for this underserved population and to evaluate combined or sequential approaches based on tumor immunogenomics.

## Figures and Tables

**Table 1:** Summary of patient and tumor characteristics.

	PATIENT 1	PATIENT 2	PATIENT 3
Age	70–79 years	70–79 years	70–79 years
Comorbidities & Frailty Status	ECOG PS 2, Heavy smoker; T2DM, PVD, IHD, AF, COPD;	ECOG PS 1, Former smoker; Emphysema, COPD, Colon diverticulosis;	ECOG PS 0, History of Papillary Thyroid Ca & Hysterectomy;
Tumor Site & Histology	Anorectal SCC Colon (hepatic flexure) AC Left Lung SCC	Distal Ascending Colon AC Right Lung AC	Gastric Angulus AC Right Colon Mucinous AC
Clinical TNM & Stage	cT3N2M0 (Stage III) cT3N0M0 (Stage IIB) cT2N2M0 (Stage III)	cT3N2M1 (Stage IV) cT2aN0M0 (Stage IB)	uT4N2M0 (Stage III) cT3N0M0 (Stage IIB)
MMR Protein Loss/TMB/PD-L1 & Biomarkers	Not tested dMMR (MLH1/PMS2 loss), BRAF V600E; PD-L1 CPS 20 MMR not tested, PD-L1 TPS 3%	dMMR (MSH6 loss) MMR not tested, KRAS G12C; PD-L1 TPS 50%	dMMR (isolated PMS2 loss), HER2 negative; PD-L1 CPS 1 dMMR (MLH1/PMS2 loss), BRAF V600E
Regimen & No. of Cycles	Carboplatin + Paclitaxel + Pembrolizumab (4 cycles), followed by Pembrolizumab maintenance (11 cycles total)	Pembrolizumab monotherapy (30 cycles)	Pembrolizumab monotherapy (79 cycles)
Local Treatment	SBRT to residual lung lesion	SBRT to lung lesion	None
Best Response per Site & Assessment	cCR (PET/CT & Endoscopy)	PR (PET/CT & Colonoscopy) in Colon and Lymph node, CR in Liver & PR in lung (CT scan)	cCR (EGD & Biopsy; new local NET G1 noted) Residual adenoma with HGD (Colonoscopy/PET)
Immune-Related AEs & Grade	G3 Interstitial Pneumonitis, G2 Diarrhea	Well tolerated	Well tolerated
Treatment Status	Discontinued due to irAEs	Ongoing	Ongoing
Follow-Up Duration	3 years	20 months	4, 5 years
Survival Status	Alive, NED	Alive with SD	Alive with SD

ECOG PS: Eastern Cooperative Oncology Group Performance Status; T2DM: Type 2 Diabetes Mellitus; PVD: Peripheral Vascular Disease; IHD: Ischemic Heart Disease; AF: Atrial Fibrillation; COPD: Chronic Obstructive Pulmonary Disease; SCC: Squamous Cell Carcinoma; AC: Adenocarcinoma; SBRT: Stereotactic Body Radiation Therapy; cCR: Clinical Complete Response; irAEs: immune-related Adverse Events; NED: no Evidence of disease; SD: Stable Disease; PR: partial response; EGD: esophagogastroduodenoscopy; NET: Neuro-Endocrine Tumor; HGD: High-Grade Dysplasia.

## Data Availability

The original contributions presented in this study are included in the article. Further inquiries can be directed to the corresponding author.
